# Evaluating the Effectiveness of *Phellodendron Amurense Ruprecht* Extract as a Natural Anti-Caries Material

**DOI:** 10.3390/ph17050603

**Published:** 2024-05-08

**Authors:** Yu-Rin Kim, Gyoo-Cheon Kim, Seoul-Hee Nam

**Affiliations:** 1 Department of Dental Hygiene, Silla University, Busan 46958, Republic of Korea; dbfls1712@hanmail.net; 2 Department of Oral Anatomy, School of Dentistry, Pusan National University, Yangsan 50612, Republic of Korea; 3 Department of Dental Hygiene, College of Health Science, Kangwon National University, Samcheok 25945, Republic of Korea

**Keywords:** oral health, natural antioxidant, *Phellodendron Amurense Ruprecht*, anti-caries material, *Streptococcus mutans*

## Abstract

Background: This study aimed to investigate the antibacterial and cytotoxic potential of *Phellodendron amurense Ruprecht* (PAR) extract against *Streptococcus mutans* (*S. mutans*) and explore the possibility of using PAR extract as an anticariogenic agent. Methods: Mixed extracts were prepared at 0, 1.25, 2.5, and 5 mg/mL concentrations, and an *S. mutans*-containing solution of 100 μL was inoculated into the medium. The survival rate of human keratinocyte (HaCaT) cells was assessed to confirm stability. One-way ANOVA was performed to evaluate the antibacterial activity against *S. mutans* and the proliferation of HaCaT cells. Results: Higher concentrations of the PAR extract showed more growth inhibition of *S. mutans* over time, with the complete inactivation of *S. mutans* at 5 mg/mL. HaCaT cell density was reduced at a PAR extract concentration of 1.25 mg/mL, but IC50 was not observed, confirming that the concentration used did not affect the cytotoxicity and proliferation. Conclusions: Results showed that the PAR extract was excellent as a natural substance with anticariogenic effects that inhibited the growth of *S. mutans* and did not affect the cell viability, thus indicating the potential for clinical application.

## 1. Introduction

Dental caries is a chronic disease that occurs frequently in children and adults, causing oral health problems due to tooth loss, which affects an individual’s quality of life. The Ministry of Health and Welfare of Korea conducts a survey on children’s oral health every three years, but the permanent tooth decay experience index decreases every year It has recently stagnated, and the DMFT index surveyed in 2018 is 1.84 in Korea, which is still higher than that of advanced countries such as the OECD [1].

Dental caries is a major cause of tooth loss, and, as a result, the economic burden for functional and aesthetic recovery increases and affects systemic health. Periodontal diseases, including dental caries, are associated with cardiovascular diseases such as heart disease and stroke, and may increase or exacerbate the risk of developing diabetes [2]. In addition, the risk of respiratory infections increases, along with complications of pregnancy, and the possibility of causing cognitive impairment such as Alzheimer’s disease has been suggested [3].

As a result of the long-term research on the etiology of dental caries, the concept of the disease has been continuously changing. In the past, dental caries was considered an infectious disease caused by pathogenic bacteria. As a representative causative agent*, Streptococcus mutans* (*S. mutans*) is a bacterium that is transmitted from the mother at birth and causes caries. Like other infectious diseases, it eliminates pathogens, Efforts have been made to prevent human-to-human transmission [4]. However, in recent years, excessive sugar is supplied to the microbial group, a form of biofilm that survives in various ways in the oral cavity, resulting in an ecological imbalance in which acid-producing bacteria are converted into dominant species. Accordingly, it is understood that, as the acidity in the oral cavity changes, the process of tooth hard tissue decoction occurs, and, eventually, dental caries occur [5].

Because the inorganic loss of dental hard tissue increases in proportion to the number of highly acidic *S. mutans*, on the contrary, if the biofilm is composed of bacteria such as non-*S. mutans* and *Actinomyces*, which are advantageous for obtaining minerals, it is a more advantageous condition for obtaining the dynamic stability of minerals [6]. According to the recent hypothesis that a keystone pathogen, which plays an important role in the ecosystem, can control the ecological role of other bacteria, *S. mutans* is considered a key pathogen that promotes the growth of other acid-producing bacteria, resulting in an imbalance in oral microbial groups.

These bacteria organize themselves to form a biofilm through cell–cell interactions or connecting with other components, such as polysaccharides, proteins, and DNA, present in the medium [7]. *S. mutans* uses the available sugar to produce a sticky glycoprotein, forming dental plaque; thus, plaque formation begins with the ingestion of sucrose-containing products [8]. *S. mutans* produces the enzyme glucosyl transferase, which is involved in glycolysis [9]. This enzyme adds more glucose molecules to form dextran, which has a structure very similar to that of starch amylase. Dextran attaches firmly to tooth enamel and other bacteria, forming dental plaque [10]. Additionally, it produces lactic acid through glycolysis under anaerobic conditions and lowers the pH level, leading to the dissolution of calcium phosphates from tooth enamel [11].

Caries is a microbe-mediated disease, and mouthwashes containing fluoride, chlorhexidine, triclosan, cetylpyridinium chloride, and chlorophyll are commonly used for caries control. However, bacteria have evolved to survive in various environments and develop resistance to antibacterial reagents [12]. Reports have suggested the development of fluorine-resistant strains [13] and drug resistance against fluoride and chlorhexidine [14]. Therefore, in the case of chlorhexidine, which is most frequently used in dental clinical practice, its use should be limited to a short-term use in the high-risk group of caries with poor oral hygiene or severe dry mouth [15]. In addition, essential oils with antibacterial properties are also used in mouthwashes and toothpastes, but more research is needed on the effectiveness and safety of long-term use [16]. Recent studies have shown that antibacterial peptides (AMPs) destroy bacterial cell membranes and inhibit cell wall synthesis to exhibit extensive antibacterial activity against various bacteria, including *S. mutans*, but more research is needed on the mechanism and safety of AMPs [17]. In addition, a quorum-sensing inhibitor (QS), developed to interfere with the function of quorum sensing (QS), a microbial signal material delivery system, is reported to reduce the formation of biofilms by interfering with the communication between bacteria or decomposing autologous derivative enzymes, but research on safety is needed [18]. Chemical antimicrobials are widely used as disinfectants but with a poor regulation of biofilms and virulence factors. Notably, new compounds are necessary for the preventive treatment of dental caries because current therapeutic measures target both pathogens and commensal bacteria [12,13,14,15,16,17,18]. Thus, due to the increased bacterial resistance and side effects of antibacterial agents, research in the recent decade has shifted its focus to natural medicine [19,20,21,22,23,24].

Extracts of plant origin are generally recognized as safe by the US Food and Drug Administration (FDA) and suitable for clinical application. These natural extracts can improve the efficacy of antibiotics against bacterial pathogens [19]. *P. crocatum* Ruiz and Pav contain secondary metabolites, such as essential oils, flavonoids, alkaloids, and phenolic compounds, with antibacterial efficacy against *S. mutans* [20]. Curcumin, a natural compound from ginger, effectively inhibits plaque-biofilm-associated cavities by inhibiting the formation of *S. mutans* biofilm [21]. Kaempferol is a type of flavonoid found in various plants and foods, such as kale, beans, tea, spinach, and broccoli, and its effect on inhibiting *S. mutans* has been confirmed [22]. Polyphenolic extracts from Chilean propolis exhibit antibacterial and antibiofilm effects against *S. mutans* at low concentrations [23]. Embelin can inhibit bacteria that cause periodontitis and tooth decay [24]. Thus, identifying natural medicines for effective oral antibacterial agents is a significant topic deserving continual attention from the research community.

This study used peeled and dried stem barks of *Phellodendron amurense Ruprecht* (PAR), a plant of the *Rutaceae* family growing wild in Japan, China, and all regions of Korea. PAR **i**s a potent anti-inflammatory constituent of prescriptions for treating inflammatory diseases [25]. In Korea, PAR is a traditional herbal medicine used since the Shilla Dynasty, with widespread applications in detoxification, antipyretics, jaundice, diarrhea, inflammation, pneumonia, and cold [26]. PAR contains several alkaloids, such as berberine, palmatine, jatrorrhizine, and obacunone, and its anti-ulcer, immune-stimulatory, anti-oxidative (in vitro and in vivo), anti-inflammatory, anti-diabetes, and neuroprotective activities have recently been studied [27]. Additionally, it has been studied for skin melanin biosynthesis inhibitory activity [28] and lipolytic activity [29]. Reportedly, PAR shows antibacterial activity against oral bacteria. In particular, it demonstrated stronger antibacterial activity against the periodontal pathogenic bacteria, *Porphyromonas gingivalis* (*P. gingivalis*), *Streptococcus spp*., such as *Streptococcus mitis* [30]. It has also been reported that berberine, one of the active ingredients in PAR, shows antibacterial activity against oral bacteria. Particularly, it shows stronger antibacterial activity against some periodontal pathogenic bacteria, such as *P. gingivalis* and *Fusobacterium nucleatum (F. nucleatum)*, compared to other oral bacteria [31], while the use of PAR and berberine would likely inhibit the relative abundance of periodontal pathogenic bacteria in the oral microbiome and, thus, be effective for preventing periodontal disease. Based on these existing studies, PAR is being used as a toothpaste for preventing periodontal disease in Japan [32]. Although there have been numerous previous studies, there is a lack of research on the antibacterial effect of PAR on *S. mutans* and cytotoxicity to human keratinocytes (HaCaT), which are essential components of oral epithelial cells. Through this study, we conducted an analysis of the antibacterial activity of PAR extract and natural-product-based cytotoxicity to provide scientific evidence to prove its beneficial effects and safety in the oral environment. Therefore, this study aims to evaluate the biocompatibility of PAR as an oral antibacterial agent, confirm its antibacterial effect against *S. mutans* (representative bacteria of dental caries), and examine HaCaT cell changes over time to the applied concentration of PAR extract for concentration-based treatments of PAR extract. Through this study, it is intended to confirm the applicability of PAR as a preventive agent for dental caries.

## 2. Results

### 2.1. Antimicrobial Effect of PAR Extract over Time

**Figure 1:**
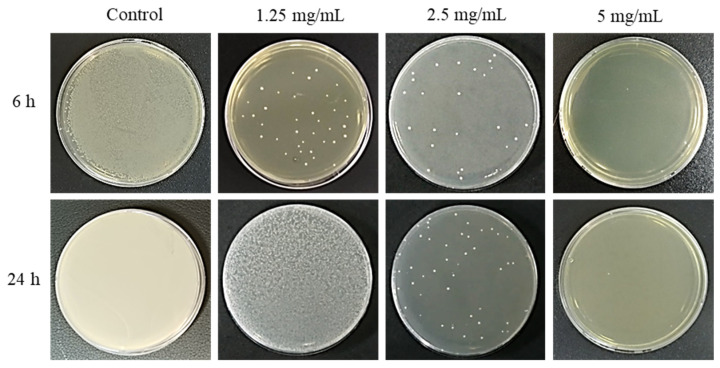
Antibacterial effect of *S. mutans* on BHI agar plate according to the concentration and application time of PAR extract.

**Figure 2:**
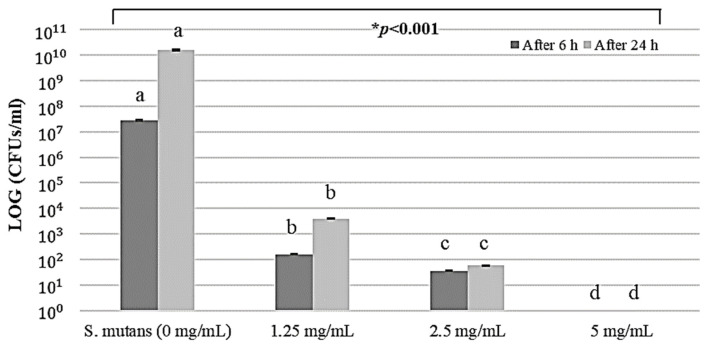
Changes and statistical analysis of CFUs of *S. mutans* according to concentration–time dependence of PAR extract. * The significant difference among the four groups in one-way ANOVA. Different letters (a, b, c, and d) by the presented statistically significant result of the post hoc Tukey’s test (*: *p* < 0.05).

The antibacterial effect of *S. mutans* over time according to the concentration of PAR extract is shown in a concentration-dependent manner, as Figure 1. The growth of *S. mutans* was inhibited with a concentration increase, with complete inactivation at 5 mg/mL. Statistical analysis of changes in CFUs of PAR extract showed a significant difference for each concentration (*p* < 0.05), with an apparent inhibitory effect of *S. mutans* (Figure 2). After 6 h, the inhibitory effect of *S. mutans* according to the concentration of the PAR extract decreased from 1.25 mg/mL and 2.5 mg/mL to 5.24 log and 5.89 log, respectively, indicating 99.99% death. At 5 mg/mL of PAR extract, all bacteria were completely killed, showing a 100% antibacterial effect. After 24 h, there was a decrease from 1.25 mg/mL and 2.5 mg/mL to 6.61 log and 8.45 log, respectively, indicating 99.99% death. PAR extract at 5 mg/mL showed a complete antibacterial effect with a 100% killing of *S. mutans* (Table 1).

### 2.2. HaCaT Cell Proliferation Activity by PAR Extract

**Figure 3:**
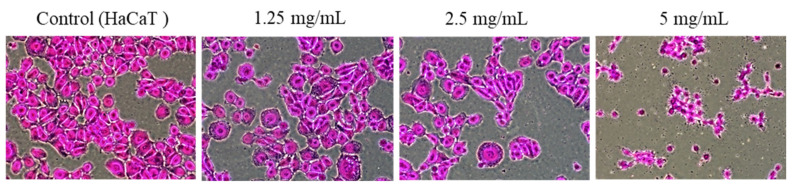
Visual evaluation through a microscope of cellular aspects through changes in HaCaT cells according to PAR extract concentration using SRB assay.

Morphological changes in HaCaT cells through PAR extract treatment of various concentrations and the SRB assay protein staining of cells resulted in a significant reduction in the number of cells and changes in cell morphology as the PAR extract concentration increased, which resulted in observable cytotoxicity associated with nucleus damage and shrinkage (Figure 3). HaCaT cells at concentrations greater than 1.25 mg/mL showed a concentration-dependent anti-proliferative effect, indicated by a lower survival with a higher growth inhibition.

### 2.3. Growth-Inhibitory Effect in HaCaT Cell

**Figure 4:**
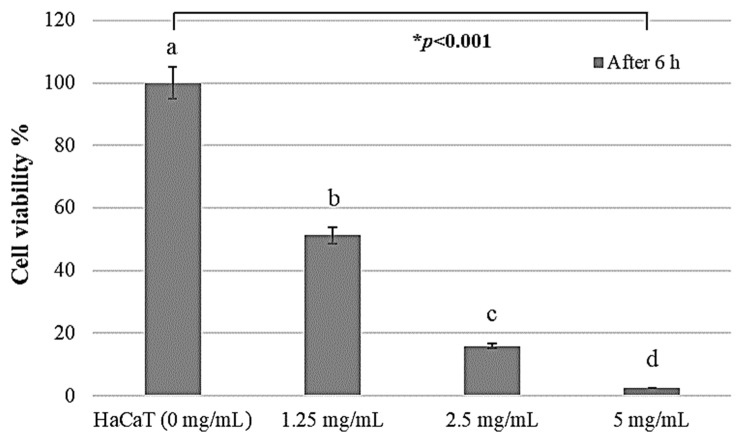
Ratio quantifying the survival rate of HaCaT cell growth treated with PAR extract using an ELISA reader at 450 nm wavelength. * The significant difference among the four groups in one-way ANOVA. Different letters (a, b, c, and d) by the presented statistically significant result of the post hoc Tukey’s test (*: *p* < 0.05).

Quantifying the cytotoxicity by PAR extract treatment by WST-1 assay and confirming the number of surviving cells, het cell viability was 51.28%, 15.95%, and 2.54% at concentrations of 1.25, 2.5, and 5 mg/mL, respectively, after 6 h (*p* < 0.05). As shown in Figure 4, PAR extract depressed the cell viability of HaCaT cells in a dose-dependent manner. The half-maximal inhibitory concentration (IC50) was 12.5 mg/mL. A higher PAR extract concentration was associated with a lower survival rate of HaCaT cells exhibiting cytotoxic activity (Figure 4). 

## 3. Discussion

As the importance of oral health has emerged due to the wearing of masks due to COVID-19, interest in managing bacteria in the mouth has increased. In addition, it was recently reported that oral diseases ranked first for three consecutive years in the 2021 Outpatient Frequent Disease Statistics of the Health Insurance Review and Assessment Service’s healthcare big data. Oral disease is a representative national disease that is more common than the common cold, and interest in oral health is increasing [33].

The high prevalence of dental caries indicates an urgent need to identify an effective, efficient, and non-toxic alternative treatment option. Treatment based on herbs derived from medicinal plants has great potential [34]. Although previous studies have confirmed the antibacterial effect of PAR extract [26,27,28,29], no study has reported its cytotoxicity. This study evaluated the antibacterial activity of PAR extract for *S. mutans*, a dental caries bacterium, and cytotoxicity using HaCaT cells.

This study showed that the growth of *S. mutans* was inhibited over time as the concentration of PAR extract increased, with the complete inactivation of *S. mutans* at 5 mg/mL. This result is similar to the research results of Kwak et al. [35], and it was reported that the higher the extraction temperature and extraction time of the PAR extract, the greater the growth inhibition effect of *S. mutans*. Studies show that PAR peels have antibacterial activity due to the presence of compounds, such as alkaloids, limonoids, phenolic compounds, quinic acid, lignans, and flavonoids [26,27,28,29]. Thus, the antibacterial effect of PAR extract could be related to these components. In contrast, Kim amd Jo [36] reported that PAR extract had no antibacterial effect on *S. mutans* and other oral strains. Therefore, there is a need for additional verification based on experimental results using multiple oral strains by varying PAR extract concentrations and clinical studies with subjects who have actual oral cavities.

Previous studies confirmed the antibacterial activity of natural substances, such as plant extracts, against *S. mutans* [37,38]. Magnolol and honokiol extracted from magnolia bark exhibited bactericidal and anti-biofilm activity against *S. mutans* [38], and some other extracts showed antibiofilm activity mainly due to the inhibition of competence-stimulating peptide (CSP) or electroporation [38]. PAR extract should exhibit a low cytotoxicity to normal oral cells to enable clinical use for their antibacterial effect against *S. mutans*. This study evaluated the cytotoxicity of PAR extract by examining morphological changes in HaCaT cells stained with cell proteins through an SRB assay. Drug cytotoxicity and cell proliferation for drug screening were measured by evaluating morphological changes using the SRB assay based on binding to cellular proteins [39]. HaCaT cells showed a significantly reduced concentration due to the PAR extract treatment, with significant morphological changes observed at higher concentrations. In contrast, cells treated with 1.25 mg/mL PAR extract maintained a constant shape and size, although the number of cells decreased and cell adhesion was weakened (Figure 3). These findings indicate that cell damage occurred with PAR extract treatment at a concentration of 1.25 mg/mL or higher, causing a significant decrease in the viability of HaCaT cells.

Additionally, the study used a WST-1 assay to indicate cell survival and growth. The WST-1 assay is typically used to determine the presence of live cells with mitochondria or detect cytotoxicity and cell growth. As a result, for an over 1.25 mg/mL concentration, the proliferation inhibitory effect was present in a concentration-dependent manner, implying that HaCaT cells appeared to show a higher growth inhibition and a lower chance of survival. Similarly, Jung et al. [40] found that cytotoxicity was evidenced at a concentration of 25 μg/mL, confirming the inhibition of cell proliferation. In subsequent experiments, they reported that the treatment concentration of the yellow-white fermented product was not toxic to cells at 12.5 μg/mL, which is identical to the findings of our study. This study found that 1.25 mg/mL PAR extract concentration reduced HaCaT cell density by using WST-1 assay to quantify cytotoxicity by PAR extract treatment and determining the number of viable cells; however, IC50 was not shown at this concentration, not affecting cytotoxicity and cell proliferation. Thus, 1.25 mg/mL of PAR extract for *S. mutans* was confirmed to be a concentration inhibiting bacterial growth with an anticariogenic effect that does not affect cell viability. Jung et al. [40] found that HaCaT cells were not toxic at a 12.5 μg/mL concentration in their evaluation of XTT cytotoxicity; this concentration used is identical to our study. Nevertheless, the authors tested this with the fermented product inoculated with the Lactobacillus plantarum CM bacteria, which differed from this study that used *S. mutans.*

The SRB analysis results indicated that PAR extract may affect the cell attachment and morphology of HaCaT cells in a dose-dependent manner. Similarly, PAR extract significantly reduced cell viability in a dose-dependent manner. Following treatment with PAR extract for 6 h, cell viability was significantly decreased in the HaCaT cells, as examined by the SRB and WST-1 assays (Figure 3 and Figure 4). These results show that PAR extract has a dose-dependent cytotoxicity for most oral epithelium.

A recent report by Okuda T et al. [41] confirmed the effect of PAR by culturing saliva-derived microbial communities. Their study showed a significant decrease in the relative abundance of red and orange complex bacteria, especially other periodontal pathogenic bacteria such as *P. gingivalis*, *F. nucleatum*, *F. periodonticum,* and *Campylobacter. showae*. In addition, the number of operational taxonomic units, the Shannon diversity index, and the Simpson diversity index, which represent the diversity of the microbial community, decreased as the PAR concentration increased. As such, PAR has the ability to regulate the structure of oral micro-organisms and has anti-inflammatory effects, suggesting that it can be helpful in preventing periodontal disease and dental caries.

This study has several limitations. First, our results cannot be generalized as our experiment was conducted in a controlled laboratory environment. The room temperature in the laboratory environment may not have been identical to the oral temperature. Moreover, PAR extract was in continuous contact with *S. mutans* in the culture medium or in vitro. In contrast, when used as a mouthwash, it would be quickly diluted and neutralized in the mouth. Additional studies are needed to elucidate the antibacterial effects of PAR extract and its potential adverse effects on the oral microflora for consideration as a mouthwash and used in vivo. Second, the anticariogenic effect of PAR was confirmed by limiting it to *S. mutans* among various dental-caries-causing bacteria. In future studies, various caries-causing bacteria should be identified, and not only the anticariogenic effect, but also the antibacterial effect against bacteria related to periodontal disease or other oral diseases, should be investigated. Third, the safety of the PAR extract was not secured through more diverse normal cells. In order to use it as an anticariogenic agent in dental clinical practice, safety must be sufficiently secured through various normal cells. Accordingly, it is necessary to identify various cells such as human normal oral keratinocytes, human gingival fibroblasts, and B16 F10 mouse melanoma cells used in cytotoxicity experiments to ensure safety. Lastly, after the safety of PAR is sufficiently secured, it is necessary to confirm the safety and applicability of long-term use through animal and human experiments. There is a need to prove its safe effectiveness through verification based on clinical indicators of its role as an anti-caries agent in the human oral environment.

Through this study, we found that 1.25 mg/mL of PAR extract inhibited the growth of *S. mutans* but did not affect cell viability. In particular, it is significant in that it evaluated not only the simple antibacterial effect of the PAR extract but also its practicality for clinical application by evaluating the cell viability of human keratinocytes (HaCaT), normal cells in the oral cavity. Therefore, we confirmed the clinical usefulness and applicability of PAR extract as an anti-caries natural substance. In the future, not only the evaluation of the antibacterial activity of PAR, but also the total polyphenolic compounds, total flavonoids, and DPPH radical scavenging activity showing antioxidant power should be confirmed. In addition, in order to identify various pathways that suppress the growth of *S. mutans*, more specific analysis using RNA-seq to confirm changes in gene expression will be needed. In order to apply PAR clinically, securing the sufficient stability of PAR should be given priority. Therefore, studies to confirm safety with various normal cells such as keratinocytes in gingival tissue should be conducted, and animal tests should be conducted first before clinical trials.

## 4. Materials and Methods

### 4.1. Phellodendron amurense Ruprecht (PAR) Extract

PAR was purchased from Hwalim Natural Drug Co., Ltd. (Busan, Republic of Korea); 70% ethanol was added to 100 g of crushed PAR, and the extraction was processed in a heating mantle at 60 °C for 12 h. Concentrated PAR was acquired using a rotary vacuum evaporator (N-1300E.V.S. EYELA Co., Tokyo Rikakikai Co., Ltd., Tokyo, Japan) after filtering it with filter paper. PAR was lyophilized using a freeze dryer (Ilshin Lab Co., Yangju, Republic of Korea) to obtain the powder. The powdered sample was stored at −20 °C after dilution.

### 4.2. Bacterial Strain

*S. mutans* (KCTC 3065/ATCC 25175) was incubated in a brain–heart infusion broth (BHI broth; Sigma-Aldrich, St. Louis, MO, USA) and cultivated at 37 °C for 24 h. The cultured bacteria were inoculated, each with 1 × 10^5^ colony-forming units per milliliter (CFU/mL).

### 4.3. Antibacterial Activity

The antibacterial effect of each PAR extract was measured by preparing mixed extracts at 1.25, 2.5, and 5 mg/mL concentrations. An *S. mutans*-containing solution of 100 μL was inoculated into the medium, cultured anaerobically, and incubated at 37 °C for 24 h. The mixture was diluted to spread a precise amount onto a BHI agar medium. Each tube was uniformly smeared on a BHI agar plate, and then cultured at 37 °C for 6 and 24 h to check the number of CFUs.

### 4.4. Cell Culturing and Identifying the Patterns of Cell Growth

HaCaT cells used in this experiment were purchased from the Department of Oral Anatomy, Pusan National University School of Dentistry (Yangsan, Republic of Korea). HaCaT cells were cultured in Dulbecco’s modified Eagle’s medium (DMEM, Gibco^®^, Waltham, MA, USA) supplemented with 10% fetal bovine serum (FBS, HyClone^®^, Logan, UT, USA) and 1% antibiotics (penicillin/streptomycin, Gibco^®^). Subculture was performed every other day at 37° C and 5% CO_2_ conditions to maintain a monolayer of cells.

Sulforhodamine B (SRB) assay was performed on the cells [42]. HaCaT cells (1 × 10^4^ cells/mL) were seeded onto 24-well plates, incubated in a 37 °C, 5% CO_2_ incubator for 24 h, diluted with PAR extracts of 0, 1.25, 2.5, and 5 mg/mL concentration in the DMEM medium, and cultured for 6 h. After incubation, we removed the medium and fixed the cells with 500 μL of 4% paraformaldehyde (Sigma-Aldrich, St. Louis, MO, USA) for 30 min at room temperature. After removing paraformaldehyde, the cells were washed multiple times with tap water and stained with 0.4% SRB (Sigma-Aldrich, St. Louis, MO, USA) solution according to the standard SRB analysis protocol. After removing the SRB solution, the plate was washed with 1% acetic acid (Daejung, Seoul, Republic of Korea) to remove the SRB dye and allowed to dry completely. Finally, an optical microscope (OLYMPUS Optical Co., Melville, NY, USA) was used with 100× magnification to examine the cells and generate the images.

### 4.5. Cell Cytotoxicity Evaluation

The effect of PAR extract on the cell growth rate was quantified using a water-soluble tetrazolium salt (WST-1) analysis using EZ-Cytox Cell Viability Assay Kit reagent (DoGenBio, Suwon, Republic of Korea) mixture [32]. HaCaT cells (1 × 10^4^) were dispensed onto a 96-well plate and cultured for 24 h. PAR extract was diluted in DMEM medium to 0, 1.25, 2.5, and 5 mg/mL concentrations. After culturing the treated cells for 6 h, WST-1 solution was applied to each well and placed in a 37 °C, 5% CO_2_ incubator for 2 h for reactions to occur. Subsequently, cell viability levels were compared by measuring absorbance at a wavelength of 450 nm using an ELISA reader (Multiskan FC, ThermoFisher Scientific, Waltham, MA, USA). All experiments were conducted three times independently for the significance of the results.

### 4.6. Statistical Analysis

Statistical software (SPSS v.26.0, SPSS Inc., Chicago, IL, USA) was used to evaluate antibacterial activity and cell growth rate of each PAR extract concentration through a one-way analysis of variance (one-way ANOVA) and post hoc Tukey’s test at a significance level of 0.05.

## 5. Conclusions

For *S. mutans*, the causative bacterium of dental caries, the density of HaCaT cells decreased at a 1.25 mg/mL concentration of PAR extract, with no significant changes in cell morphology; thus, the concentration used did not affect cytotoxicity and cell proliferation. Thus, PAR extract at a 1.25 mg/mL concentration exhibits an anticariogenic effect and inhibits bacterial growth without affecting cell viability for *S. mutans*. PAR extract, used in appropriate concentrations, shows anticariogenic potential and can be of high value as a natural biocompatible anticariogenic agent. 

## Figures and Tables

**Table 1:** Differences in quantitative bacterial count and growth of *S. mutans* according to change in concentration of PAR extract and time.

	Control (*S. mutans*)	1.25 mg/mL	2.5 mg/mL	5 mg/mL
After 6 h	2.8 ± 0.5 × 10^7^	1.6 ± 0.2 × 10^2^	3.6 ± 0.3 × 10^1^	0.0
After 24 h	1.6 ± 0.6 × 10^10^	4.0 ± 0.9 × 10^7^	5.7 ± 0.4 × 10^1^	0.0

## Data Availability

The data presented in this study are available on request from the corresponding author.
